# Decentralization and Regionalization: Redesigning Health Systems for High Quality Maternity Care Comment on "Decentralization and Regionalization of Surgical Care: A Review of Evidence for the Optimal Distribution of Surgical Services in Low- and Middle-Income Countries"

**DOI:** 10.34172/ijhpm.2020.30

**Published:** 2020-03-03

**Authors:** Sanam Roder-DeWan

**Affiliations:** UNICEF, Health Section, Dar es Salaam, Tanzania.

**Keywords:** Regionalization, Decentralization, Service Delivery Redesign, Health System Quality, Maternal Health

## Abstract

The question of how to optimally design health systems in low- and middle-income countries (LMICs) for high quality care and survival requires context-specific evidence on which level of the health system is best positioned to deliver services. Given documented poor quality of care for surgical conditions in LMICs, evidence to support intentional health system design is urgently needed. Iverson and colleagues address this very important question. This commentary explores their findings with particular attention to how they apply to maternity care. Though surgical maternity care is a common healthcare need, maternal complications are often unpredictable and require immediate surgical attention in order to avert serious morbidity or mortality. A discussion of decentralization for maternity services must grapple with this tension and differentiate between facilities that can provide emergency surgical care and those that can not.

Iverson and colleagues tackle a critically important topic that remains understudied in low- and middle-income countries (LMICs): what is the optimal way to organize a health system to produce good surgical outcomes^1^? Though the evidence base for answering this question in LMICs is anemic, a mounting body of work suggests that improving the quality of surgical care needs urgent attention.^2,3^ How one organizes a health system can impact outcomes across a variety of conditions; intentional and outcomes-oriented health system design should be considered a core health reform by countries struggling with poor quality of care. The Lancet Global Health Commission on High Quality Health Systems identifies service delivery redesign as one of four universal actions that LMICs should consider when strategizing to improve quality.^4^ Service delivery redesign means restructuring where and by whom services are delivered to ensure the delivery of high-quality care. Conditions that require advanced training, multi-disciplinary care teams, and high technological inputs should ideally be shifted to centralized hospitals, while services that would benefit from closeness to community, are more chronic and need many health service contacts should be delivered in more distal primary care facilities.^5^ Iverson et al contribute to the evidence-base for how best to implement service delivery redesign.

Health systems in many LMICs are inherited from an epidemiological era dominated by communicable diseases. These systems were designed to deliver episodic care to high mortality populations, and in many cases, for conditions that were funded vertically through development partnerships.^6^ Though we are far from being able to say that communicable diseases are behind us,^7^ impressive immunization coverage, improved public health measures and the successful scaling of relatively simple treatments for common illnesses (eg, oral rehydration for childhood diarrhea), have meant that the health systems of today are left with a different, and more complex, mix of challenges.

Systems were also designed to maximize access. Global attention is now turning to the long-neglected role of quality of care in improving outcomes.^8^ Accessing services is not enough to produce good health if those services do not meet minimum standards of quality.^9,10^ In a study of mortality due to poor quality of care, Kruk and colleagues estimated that in 2016, 5 million excess deaths globally were due to poor quality of care.^11^ How should health systems change to respond to the changing burden of disease? Where, and by whom, should services be delivered to maximize system assets and produce the best outcomes for people?

In their paper, *Decentralization and Regionalization of Surgical Care: A Review of Evidence for the Optimal Distribution of Surgical Services in Low- and Middle-Income Countries*, Iverson et al grapple with one of the core dimensions of service delivery design, regionalization vs. decentralization.^1^ Though, as they state, strong evidence from high-income countries exists and points to the benefits of regionalization for rare, high-complexity conditions, it is unclear which conditions are best treated where and by whom in LMICs. They look specifically at surgical conditions, including obstetric procedures, through a scoping review of the literature. They conclude that decentralization, especially for obstetric procedures, has been successful in the majority of studies and go on to recommend criteria for selecting the best health system level for a particular service.

A few key issues should be considered when interpreting their results, especially as they apply to obstetric care. First, the authors have excluded natural childbirth from their analysis. Unlike other surgical conditions where the need for surgery is often immediately apparent and dictates triage decisions upon patient presentation, childbirth is an exception. The need for surgical intervention is often unknown when a pregnant woman presents to the health system. When surgery is needed, the need often arises quickly in patients that may have been considered low-risk initially. For example, studies from a variety of settings suggest that about one third of women categorized as low-risk will go on to develop a complication requiring advanced care.^12,13^ By excluding studies that look at vaginal deliveries, it becomes difficult to understand where obstetric services are best delivered overall.

Another key methodological issue should be considered when interpreting the the literature. The authors make the practical decision to use two health system levels to organize and analyze the literature. The first category – “decentralized” – includes health centers and district hospitals. The second category includes regional hospitals and higher. Though perhaps logical and even clinically sound for the analysis of many conditions, obstetrics is an exception. Recent evidence shows that secondary facilities with cesarean section capacity deliver higher quality intrapartum care than primary care facilities.^14^ The important dividing line for obstetrics appears to be whether or not the facility can deliver high quality advanced obstetric and neonatal care (ie, *comprehensive emergency obstetric and newborn care*), not whether it is above or below the level of a regional hospital. Decentralizing to a health facility without functional surgical services or access to blood products is very different from decentralizing to a district hospital capable of dealing with an obstetric or neonatal emergency, but these scenarios are considered jointly in the Iverson review. Gabrysche and colleagues illustrate this point nicely in their study of 119 244 pregnancies in Ghana where they show that access to advanced high-quality care leads to improved outcomes, not access to any facility for delivery.^15^ The categorization decision made by Iverson et al masks this important distinction and makes their results difficult to interpret and apply to obstetric system design efforts in LMICs.

Finally, though the authors do not intend to directly compare regionalization to decentralization, and indeed do not review studies that do so, the structure of the paper may suggest that their findings are robust to comparison. In reality, and as the authors clearly state, each included study compares an intervention to “business-as-usual.” Unfortunately, business-as-usual at all levels of the health system in LMICS often means poor quality of care, poor provider performance, system shortcomings and poor outcomes.^4,16^ Interventions that bring resources and attention can often improve processes of care in the short-run, but sustaining improvements is the perennial challenge. The question of how to restructure health systems for optimal outcomes cannot be adequately answered without understanding the sustainability of proposed changes. The field sorely needs longitudinal studies that explore structural improvements to health systems and follow their impact over longer periods of time.

The authors conclude that three factors should be considered when deciding where surgical conditions should be delivered in LMIC health systems: acuity, volume and complexity. This is a valuable conclusion that countries should consider when making health system decisions about surgical services. Volume and quality are closely related. A growing literature from primarily high-income countries suggests that higher volume facilities produce better maternal and neonatal outcomes. In a study of volume and quality of obstetric care in 5 LMICS, facilities performing over 500 deliveries each year were better prepared to deliver high quality care.^10^ Without concentrating services for rare conditions in a limited set of specialized facilities, providers will not be able to acquire and maintain the skills necessary to deliver high quality care. Similarly, centralized facilities with a mix of specialized providers are more likely to be able to deliver the interdisciplinary care that is often needed to address complex conditions. Obstetric fistula surgery is a good example; where the best outcomes occur in centers that can perform surgical procedures, and provide “wrap around” care that addresses the psychological and social needs of women recovering from obstetric fistula. Acuity is a more complex consideration that requires high quality services to be closer to where people fall ill. In general, low volume, low acuity and high complexity conditions are amenable to regionalization, while high volume, high acuity, low complexity conditions might best be decentralized.

Unfortunately, low acuity, low volume and high complexity conditions do not always occur together. For example, the high acuity clinical challenge of head trauma requiring surgical intervention is complex and relatively rare. Though Iverson et al state that cesarean sections are “low resource and low complexity,” there is no consensus around this categorization, and many would argue that, though common (high volume), obstetrics is highly complex. The same obstetric interventions that can save lives, when applied in the wrong patient, can lead to unnecessary complications.^17^ Additionally, providers must have the skills to transition quickly from gently supporting a physiologic process to decisively handling life-threating pathologies. It is examples such as these where acuity, volume and complexity fail to run together that will tax health systems the most to consistently deliver high quality care to all. Surgical disparities are likely to remain the longest for these types of conditions.

The work by Iverson and colleagues is a welcome addition to the literature on health and quality in LMICs because it takes the health system as its unit of analysis on quality. This is relatively rare in the literature on quality of care which is dominated by studies that look at the “micro” level – studies that analyze the user interaction with health facilities, the behaviors of providers or facility operations. An analysis of the primary care improvement literature estimate that about three quarters of published studies targeted this micro level.^4^ Though measurement of quality and efforts to improve quality are necessary at all levels of the health system, the literature is incomplete without an understanding of the macro and meso levels. More importantly, the Lancet Global Health Commission on High Quality Health Systems argues that improvement at the meso and macro levels through structural reforms stand to make the biggest impact in settings that need big changes to consistently deliver high quality care.^4^ The study by Iverson et al is the type of analysis needed to begin informing these structural reforms and moving LMICs towards high quality health systems.

## Ethical issues

Not applicable.

## Competing interests

Author declares that she has no competing interests.

## Disclaimer

The views expressed in this article are my own and not those of UNICEF.

## Author’s contribution

SRD is the single author of the paper.
